# Accuracy of Brain Computed Tomography Diagnosis by Emergency Medicine Physicians

**DOI:** 10.1155/2022/5659129

**Published:** 2022-09-26

**Authors:** Zohair Al Aseri, Mohamed Al Aqeel, Badr Aldawood, Fahad Albadr, Rawan Ghandour, Abdulaziz Al Mulaik, Mohammed A. Malabarey, Anas Khan

**Affiliations:** ^1^Department of Emergency Medicine, College of Medicine, King Saud University Medical City, King Saud University, Riyadh, Saudi Arabia; ^2^Department of Critical Care, College of Medicine, King Saud University Medical City, King Saud University, Riyadh, Saudi Arabia; ^3^Radiology and Medical Imaging, Neuroradiology, King Saud University Medical City, King Saud University, Riyadh, Saudi Arabia

## Abstract

**Objectives:**

The objective of this study is to prospectively analyze emergency physicians' (EP's) abilities to interpret noncontrast computed tomography (NCCT) brain images in a blinded fashion and assess whether they can make medical decisions solely based on their interpretations.

**Methods:**

A cross-sectional study was conducted at the emergency department (ED), King Saud University Medical City (KSU-MC), Saudi Arabia, over a period of one year, from May 2014 to May 2015. Any patient who underwent plain brain NCCT during the study period in our ED was included in this study. An independent attending neuroradiologist compared the EP's interpretations with the official final reports dictated by an on-call radiologist.

**Results:**

A brain NCCT prospective chart audit of 1,524 patients was interpreted by ED physicians (EP) at KSU-MC from 2014–2015. The ages of patients were between 14 and 107 years, and the mean ± SD age was 45.6 ± 22.1 years. Radiological brain lesions were confirmed by EPs and radiology physicians in 230 (15.09) and 239 (15.68) patients, respectively, out of which concordance was observed in 170 (71.13) cases, with a kappa value of *r* = 0.675. Normal, chronic, and nil acute reports were made by EPs and radiology physicians for 1,295 (84.97) patients and 1,285 (84.32) patients, respectively, out of which concordance was observed in 1,225 (95.33) cases, with a kappa value of *r* = 0.672. The study results demonstrated that the overall agreement between EPs and radiologist specialists was 91.6, with a kappa value of .675 (*p* < 0.001).

**Conclusion:**

Emergency physicians are moderately accurate at interpreting brain NCCT compared to radiologists. More research is needed to discover the most cost-effective technique for reducing the number of significant misinterpretations.

## 1. Introduction

Neurologic and traumatic complaints are frequently screened in the ED with the use of brain NCCT, which is required in both critical and noncritical cases [1]. EPs must respond promptly to trauma and other severe situations in accordance with the findings of related investigations since time is important [2]. Many EDs operate at a fast pace to avoid the formality of referrals and tracking radiological reports to save time and provide optimal management to their patients. However, some studies have implicated diagnostic imaging as a direct cause of increased ED length of stay [3, 4]. To improve clinical decision-making and treatment planning, the use of NCCT in patients with ED conditions might be beneficial via improved diagnostic confidence in the NCCT results [5]. Patients, healthcare providers, and managers might all benefit from an EP capable of appropriately understanding NCCT [6].

The discordance between a senior EP and a consultant radiologist was recorded at 14.8%, while the discordance between a junior radiology trainee and a consultant radiologist was recorded at 40% [7]. Another study analyzed the interpretations of EPs and medical registrars and compared them to the final report of a radiologist, with a disagreement rate of 33% [8]. In a case series, the authors reached 13.4% disagreement between the interpretations of EP and two senior radiologists; however, there were no clinically relevant misinterpretations [9].

The current evidence shows inconsistent findings regarding the agreement between EPs and radiologists when it comes to brain NCCT. Therefore, in this study, we aimed to prospectively analyze EPs' abilities to interpret brain NCCT in a blinded fashion and assess whether they could make medical decisions solely based on their interpretations.

## 2. Methods

This cross-sectional study was conducted at the ED of King Saud University Medical City (KSU-MC). The study targeted 1,524 individuals who came to our ED while receiving brain NCCT and was conducted over a period of one year, from May 2014 to May 2015. Any patient who underwent plain brain NCCT during the study period in our ED was included in this study. We excluded only patients whose brain NCCT was interpreted by physicians other than EPs (on-call radiologists/admitting medical services, i.e., neurologists or neurosurgeons).

Ordering physicians are required to fill out patient identifying information, the study's initial interpretation, and the patient's final disposition plan before brain NCCT is performed. Only board-certified EPs with variable clinical experience were included in the evaluation. None of the EPs received further training or instructions for brain NCCT. After performing brain NCCT, the EP evaluated it and recorded their comments and dispositions on a form provided by a nurse. If the CT results were not documented on the EP report form when reported by the radiology expert, they were excluded from the study. Later, the forms were retrieved from the study's designated box. Finally, the radiological reports were tracked through the radiology system.

An independent attending neuroradiologist and two emergency medicine consultants compared the EP's interpretation with the official final reports dictated by the on-call radiologist. Upon reviewing each report, it was deemed either positive with no discrepancies or discrepant. Acute hemorrhage, acute/subacute infarction, evidence of space-occupying lesions, brain edema, evidence of cavernous sinus thrombosis, facial bone fracture, or acute hydrocephalus were all considered positive findings in our study since they might have altered the patient's immediate disposition. Positive NCCT with no discrepancies occurs when the ED physician and the on-call radiologist agree on the etiology. It is important to distinguish between fatal and nonfatal lesions because fatal ones need immediate attention and specialized consultation, while nonfatal ones do not. Data analysis was carried out using the Statistical Package for Social Science software (IBM SPSS Statistics Grad Pack 28.0). The sensitivity, specificity, concordance, and kappa coefficient were calculated using the radiologist's judgment as the reference standard for evaluating the inter-rater reliability between the radiologist's report and EP's impression. Excellent agreement is defined as a kappa value of >0.75, with 0.40–0.75 being considered moderate, and <0.40 being regarded as poor. A *p* value of <0.05 was considered significant.

## 3. Results

As shown in Table 1, an emergency brain NCCT prospective chart audit of 1,524 patients was interpreted by EPs at KSU-MC from 2014-2015. The patients were over 14 years and presented to the ED undergoing NCCT brain as per EP discretion. The ages of the patients were between 14 and 107 years, and the mean ± SD age was 45.6 ± 22.1 years.

The chief complaints in the patients were trauma (27.4%), loss of consciousness (23.1%), headache (22.6%), weakness/numbness (16.8%), dizziness (9.4%), seizure (8.7%), nausea and vomiting (5.9%), dysphasia, aphasia, and speech difficulty (5.0%). Visual disturbances, difficulty walking/ataxia, craniopathies, vertigo, amnesia, insomnia, and urine incontinence were recorded at 18.9%.

The indications for ordering brain NCCT were to look for intracranial bleeding (877, 57.5%), ischemic stroke (435, 28.5%), and space-occupying lesion/metastasis (197, 12.9%), while 27 (1.9%) cases were for hydrocephalus, cavernous sinus thrombosis, facial bone fracture, edema, and shift. Other 408 (26.8%). 132 (8.7%) cases had not been documented.

The ED observed 1–3 findings per patient, thereby documenting 256 lesions in 230 (15.1%) patients, while most reports (1,295, 84.9%) were considered normal, chronic, or nil acute. Similarly, radiologists had observed 1–3 findings per patient, thereby documenting 271 lesions in 239 (15.7%) patients, while most of the reports (1,285, 84.3%) were deemed normal.

As shown in Table 2, brain NCCT findings were classified into ten findings. The EP's report was tallied with the radiologist's confirmed report for each of the specified findings. Inter-rater agreement was represented by the percentage sensitivity score, and the measure of association between EPs and radiologists was agreed upon with consideration of the kappa test results.

EPs and radiologists reported intracranial bleeding in 24 (1.57) and 26 (1.71) patients, respectively, among which concordance was observed in 18 (69.23) cases, with a kappa value of *r* = 0.715. Subdural hemorrhage was reported in 20 (1.31) and 18 (1.18) patients, respectively, among which concordance was observed in 12 (66.67) cases, with a kappa value of *r* = 0.627. Subarachnoid hemorrhage (SAH) was observed in 12 (0.79) and 7 (0.46) patients, respectively, among which concordance was observed in six (85.71) cases, with a kappa value of *r* = 0.629. Ischemic stroke was reported in 120 (7.87) and 123 (8.07) patients, respectively, among which concordance was observed in 74 (60.16) cases, with a kappa value of *r* = 0.575. Space-occupying lesions were reported in 22 (1.44) and 30 (1.97) patients, respectively, among which concordance was observed in 18 (60.00) cases, with a kappa value of *r* = 0.687. Edema and shift were reported in 24 (1.44) and 20 (1.97) patients, respectively, among which concordance was observed in 14 (70.00) cases, with a kappa value of *r* = 0.631. Hydrocephalus was reported in six (0.39) and seven (0.46) patients, respectively, among which concordance was observed in three (42.86) cases, with a kappa value of *r* = 0.459. Cavernous sinuses thrombosis was reported in four (0.26) and three (0.20) patients, respectively, among which concordance was observed in three (85.71) cases, with a kappa value of *r* = 0.857. Facial bone fractures were reported in 18 (1.18) and 32 (2.10) patients, respectively, among which concordance was observed in 17 (53.13) cases, with a kappa value of *r* = 0.675.10-epidural hematoma was reported in six (0.39) and five (0.33) patients, respectively, among which concordance was observed in five (90.19) cases, with a kappa value of *r* = 0.909.

There were normal, chronic, and nil acute ED reports for 1,295 (84.97) patients and radiologist reports for 1,285 (84.32) patients, respectively, out of which concordance was observed in 1,225 (95.33) cases, with a kappa value of *r* = 0.672.

CT brain lesions were confirmed by EPs in 230 (15.09) patients and radiologists in 239 (15.68) patients, out of which concordance was observed in 170 (71.13) cases, with a kappa value of *r* = 0.675. The overall agreement between EPs and radiologist specialists was 91.6, with a kappa value of .675 (*p* < 0.001).

## 4. Discussion

For traumatic patients, accurate interpretation of NCCT brain abnormalities is critical for prompt and appropriate management. Previously, many studies have been conducted to evaluate the accuracy of EPs' interoperations with NCCT and other imaging modalities; however, the design and methodology of these studies vary considerably [10, 11]. The interpretation of plain X-rays was the main focus of most of these studies rather than CT, with an overall discordance rate ranging from 0.95% to 16.8% [12, 13]. It was reported that the total discordance rate between the interpretations of NCCT by EPs was 37.1% in the only comparable prospective study [14]. In this study, our findings showed 91.6% concordance between EPs and radiologists. The agreement between EP and radiologist specialists was good, with a kappa value of .675 (*p* < 0.001).

Similar to our findings, Khan et al.showed that the concordance between EPs and radiologists was observed in 87.14% of the interpreted NCCT brain images with good agreement (kappa = 0.64) and a high degree of accuracy of 90.5%. They also showed that the false-negative rate of EPs was 3.6% [17]. In an English retrospective study, Mucci et al. investigated the accuracy of five EPs in the interpretation of 100 NCCT brain images. Their findings demonstrated that the overall agreement was 86.6%, and the false-negative rate was 4.2% [10]. It is common to see 1%–3% false-negative rates in published studies; however, in some studies, they may reach 11%. Using a set of completely abnormal images, Vincent et al. reported that 35% of EPs made mistakes [19]. Nevertheless, clinical outcomes are rarely affected by missed diagnoses during the time between initial interpretation and radiological evaluation [17–19].

In an Australian study, the authors demonstrated that EPs were able to accurately interpret 85.20% of cases, with a kappa value of 0.69. Out of discordant events, 41% of cases appeared with potential or definitive complications. Among the scans that the radiologist deemed abnormal, the discordance rate was higher. They also highlighted that there was no significant difference in NCCT interpretation accuracy based on the EP's degree of experience or training [7]. The likeliest reason is that CT interpretation accuracy is mostly determined by formal training rather than emergency medical skills. Seniority does not seem to have any effect on physicians' ability to report either plain films or CT scans [19, 20]. With only one to two hours of training, EPs have shown a statistically significant increase in NCCT reporting accuracy [21]. In addition, the first year of radiology training has a large impact on trainees' accuracy, but this influence diminishes with time as variations between individuals become more apparent [22].

According to Khoo and Duffy, only around two-thirds of senior EPs' interpretations of NCCT scans are accurate. The proportion of “abnormal” scans in their study population was 26%, which yielded a decent negative predictive value of 82.3% [9]. How much precision is needed to ensure safe practice remains unclear. Even though their clinically significant misinterpretation rate was just 6.1%, Arendts et al. judged that their level of accuracy was “no better than moderately good” [8]. Alfaro et al., on the other hand, reported that despite a very high incidence of misinterpretation, just 0.6% of patients were treated improperly, and none had an unfavorable result in the chart review [16].

SAH is a life-threatening condition that commonly results in brain damage and even death [23–25]. It may be difficult to diagnose and could present with nonspecific complaints [26]. There is a greater risk of re-bleeding and associated death if SAH is diagnosed and treated late [27, 28]. Our study showed an accuracy of 99.50 for diagnosing SAH. Some studies have shown that the misdiagnosis of SAH varies between 12% and 51% [29–31]. Access to diagnostic resources, physician experience, and patient acuity are all important risk factors for misdiagnosis. A large cohort study showed that the rate of misdiagnosis of cerebral cavernous sinuses thrombosis was 3.6%, which was associated with an increased risk of a longer length of hospital stay, an unfavorable discharge disposition, intracerebral hemorrhage, and in-hospital mortality [32]. In our study, the accuracy of diagnosing cavernous sinus thrombosis was 99.90, but there were only four cases. A cross-sectional study of 2,288 cases showed that fractures were the most common conditions associated with diagnostic errors in ED (44%), followed by intracranial bleeding (6%). They also stated that human mistakes, healthcare professionals' inadequate skills or knowledge, and inability to comply with protocols were among the major factors for these errors [33, 34]. Our study showed an accuracy of 99% in diagnosing intracranial bleeding and facial bone fractures.

We acknowledge that our study has some limitations, including the single-center setting, which may induce the risk of selection bias. Therefore, multicenter studies are required to investigate the predictors of EPs' interpretations, including the variation between healthcare systems, access to diagnostic tools, compliance of physicians with the protocol, and training received [35–37].

In conclusion, our EPs were moderately accurate in interpreting brain NCCT compared to radiologists. More research is needed to discover the most cost-effective technique for reducing the number of significant misinterpretations. Brain NCCT interpretation instruction sessions may significantly enhance EPs' accuracy.

## Figures and Tables

**Table 1:** Descriptive statistics.

Characteristics	Description	*N* (%)
Age (year)	Mean ± SD	45.6 ± 22.1
	Minimum-maximum	14–107

Chief complaints	Trauma	417 (27.4)
	Loss of consciousness	352 (23.1)
	Headache	344 (22.6)
	Weakness/numbness	256 (16.8)
	Dizziness	143 (9.4)
	Seizure	133 (8.7)
	Nausea and vomiting	90 (5.9)
	Dysphasia, aphasia, speech difficulty	76 (5.0)
	Visual disturbances	45 (3.0)
	Difficulty walking/ataxia	36 (2.4)
	Craniopathies	23 (1.5)
	Vertigo	21 (1.4)
	Amnesia	10 (0.7)
	Insomnia	7 (0.5)
	Urine incontinence	3 (0.2)
	Others	140 (9.2)

Indications	Intracranial bleeding (SAH, SDH, hemorrhagic stroke, etc.)	877 (57.5)
	Ischemic stroke	435 (28.5)
	Space-occupying lesion/metastasis	197 (12.9)
	Hydrocephalus	13 (0.9)
	Cavernous sinus thrombosis	6 (.4)
	Facial bone fracture	4 (0.3)
	Edema, shift	4 (0.3)
	Others	408 (26.8)
	Not documented	132 (8.7)

EP findings	Nil	1295 (84.9)
	One	206 (13.5)
	Two	22 (1.4)
	Three	2 (0.1)
	Total findings	230
Radiology findings	Nil	1285 (84.3)
	One	211 (13.8)
	Two	24 (1.6)
	Three	4 (0.3)
	Total findings	239

**Concordance (including nil)**		**1395 (91.6)**

**Table 2:** Inter-rater agreement between the emergency physician and radiologist.

Characteristics (*N* = 1524)			Cell frequency				Measures of association					Agreement
Site	ED findings	RAD findings	ED+/RAD+	ED+/RAD−	ED−/RAD+	ED−/RAD−	Sensitivity	Specificity	PPV	NPV	Accuracy	Kappa
Intracranial bleeding	24	26	18	6	8	1492	69.23	99.60	75.00	99.50	99.10	0.715
Subdural hemorrhage	20	18	12	8	6	1498	66.67	99.47	60.00	99.60	99.10	0.627
Subarachnoid hemorrhage	12	7	6	6	1	1511	85.71	99.60	50.00	99.90	99.50	0.629
Ischemic stroke	120	123	74	46	49	1355	60.16	96.72	61.67	96.50	93.80	0.575
Space-occupying lesions	22	30	18	4	12	1490	60.00	99.73	81.82	99.20	99.00	0.687
Edema, shift	24	20	14	10	6	1494	70.00	99.34	58.33	99.60	99.00	0.631
Hydrocephalus	6	7	3	3	4	1514	42.86	99.80	50.00	99.70	99.50	0.459
Cavernous sinus thrombosis	4	3	3	1	1	1520	85.71	99.93	75.00	100.00	99.90	0.857
Facial bone fracture	18	32	17	1	15	1491	53.13	99.93	94.44	99.00	99.00	0.675
Epidural	6	5	5	1	1	1518	90.91	99.93	83.33	100.00	99.90	0.909
Normal, chronic, nil acute	1295	1285	1225	70	60	169	95.33	70.71	94.59	73.80	91.50	0.672
Overall confirmed cases	230	239	170	60	69	1225	71.13	95.33	73.91	94.70	91.50	0.675

## Data Availability

The data used to support the findings of this study are included within the article.

## References

[1] Shah H., Jarwani B., Gjjar M. (2015). Can ed physician interpret NCCT brain reliably in head injury victims’s emergency management?. *Journal of Evolution of Medical and Dental Sciences*.

[2] Rodziewicz T. L., Houseman B. H. J. (2022). *Medical Error Reduction and Prevention*.

[3] Goloback M., McCarthy D. M., Schmidt M., Adams J. G., Pang P. S. (2015). ED operational factors associated with patient satisfaction. *The American Journal of Emergency Medicine*.

[4] Ryan A., Hunter K., Cunningham K. (2013). STEPS: lean thinking, theory of constraints and identifying bottlenecks in an emergency department. *Irish Medical Journal*.

[5] Menon B. K., Demchuk A. M. (2011). Computed tomography angiography in the assessment of patients with stroke/TIA. *The Neurohospitalist*.

[6] Badr M., Al-Otaibi S., Alturki N., Abir T. (2022). Detection of heart arrhythmia on electrocardiogram using artificial neural networks. *Computational Intelligence and Neuroscience*.

[7] Greiner A. C. K. E., Institute of Medicine (US) Committee on the Health Professions Education Summit (2003). The core competencies needed for health care professionals. *Health Professions Education: A Bridge to Quality*.

[8] Arendts G., Manovel A., Chai A. (2003). Cranial CT interpretation by senior emergency department staff. *Australasian Radiology*.

[9] Khoo N. C., Duffy M. (2007). Out of hours non-contrast head CT scan interpretation by senior emergency department medical staff. *Emergency Medicine Australasia*.

[10] Mucci B., Brett C., Huntley L. S., Greene M. K. (2005). Cranial computed tomography in trauma: the accuracy of interpretation by staff in the emergency department. *Emergency Medicine Journal*.

[11] Klein E. J., Koenig M., Diekema D. S., Winters W. (1999). Discordant radiograph interpretation between emergency physicians and radiologists in a pediatric emergency department. *Pediatric Emergency Care*.

[12] Maray M., Alghamdi M., Alazzam M. B. (2022). Diagnosing cancer using IOT and machine learning methods. *Computational Intelligence and Neuroscience*.

[13] Nitowski L. A., O’Connor R. E., Reese C. L. (1996). The rate of clinically significant plain radiograph misinterpretation by faculty in an emergency medicine residency program. *Academic Emergency Medicine*.

[14] Herman P. G., Hessel S. J. (1975). Accuracy and its relationship to experience in the interpretation of chest radiographs. *Investigative Radiology*.

[15] Nolan T. M., Oberklaid F., Boldt D. (1984). Radiological services in a hospital emergency department—an evaluation of service delivery and radiograph interpretation. *Journal of Paediatrics and Child Health*.

[16] Alfaro D., Levitt M. A., English D. K., Williams V., Eisenberg R. (1995). Accuracy of interpretation of cranial computed tomography scans in an emergency medicine residency program. *Annals of Emergency Medicine*.

[17] Khan A., Qashqari S., Al-Ali A.-A. (2013). Accuracy of non-contrast CT brain interpretation by emergency physicians: a cohort study. *Pakistan Journal of Medical Sciences*.

[18] Qader Osman N. A., Al-Ziyadi S. H., Alazzam M. B., Alshawwa S. Z., Rahman M. A. (2022). Machine learning of ZnO interaction with immunoglobulins and blood proteins in medicine. *Journal of Healthcare Engineering*.

[19] Vincent C. A., Driscoll P. A., Audley R. J., Grant D. S. (1988). Accuracy of detection of radiographic abnormalities by junior doctors. *Emergency Medicine Journal*.

[20] Wardrope J., Chennells P. M. (1985). Should all casualty radiographs be reviewed?. *British Medical Journal Publishing Group*.

[21] Eachempati S. R., Flomenbaum N., Seifert C., Fischer E., Hydo L. J., Barie P. S. (2000). Alterations of preliminary readings on radiographic examinations minimally affect outcomes of trauma patients discharged from the emergency department. *The Journal of Trauma, Injury, Infection, and Critical Care*.

[22] Mayhue F. E., Rust D. D., Aldag J. C., Jenkins A. M., Ruthman J. C. (1989). Accuracy of interpretations of emergency department radiographs: effect of confidence levels. *Annals of Emergency Medicine*.

[23] Gratton M. C., Salomone J. A., Watson W. A. (1990). Clinically significant radiograph misinterpretations at an emergency medicine residency program. *Annals of Emergency Medicine*.

[24] Levitt M. A., Dawkins R., Williams V., Bullock S. (1997). Abbreviated educational session improves cranial computed tomography scan interpretations by emergency physicians. *Annals of Emergency Medicine*.

[25] Espinosa J. A., Nolan T. W. (2000). Reducing errors made by emergency physicians in interpreting radiographs: longitudinal study. *BMJ Publishing Group*.

[26] Roos Y., De Haan R. J., Beenen L. F. M., Groen R. J. M., Albrecht K. W., Vermeulen M. (2000). Complications and outcome in patients with aneurysmal subarachnoid haemorrhage: a prospective hospital based cohort study in The Netherlands. *Journal of Neurology Neurosurgery and Psychiatry*.

[27] Hijdra A., Braakman R., Van Gijn J., Vermeulen M., Van Crevel H. (1987). Aneurysmal subarachnoid hemorrhage. Complications and outcome in a hospital population. *Stroke*.

[28] Solenski N. J., Haley E. C. J., Kassell N. F. (1995). Medical complications of aneurysmal subarachnoid hemorrhage: a report of the multicenter, cooperative aneurysm study. *Critical Care Medicine*.

[29] Alazzam M. B., Al-Radaideh A. T., Alhamarnah R. A., Alassery F., Hajjej F., Halasa A. (2021). A survey research on the willingness of gynecologists to employ mobile health applications. *Computational Intelligence and Neuroscience*.

[30] Vermeulen M. J., Schull M. J. (2007). Missed diagnosis of subarachnoid hemorrhage in the emergency department. *Stroke*.

[31] Lord A. S., Fernandez L., Schmidt J. M. (2012). Effect of rebleeding on the course and incidence of vasospasm after subarachnoid hemorrhage. *Neurology*.

[32] Tang C., Zhang T.-S., Zhou L.-F. (2014). Risk factors for rebleeding of aneurysmal subarachnoid hemorrhage: a meta-analysis. *PLoS One*.

[33] Vannemreddy P., Nanda A., Kelley R., Baskaya M. K. (2001). Delayed diagnosis of intracranial aneurysms: confounding factors in clinical presentation and the influence of misdiagnosis on outcome. *Southern Medical Journal*.

[34] Kowalski R. G., Claassen J., Kreiter K. T. (2004). Initial misdiagnosis and outcome after subarachnoid hemorrhage. *JAMA. United States*.

[35] Mayer P. L., Awad I. A., Todor R. (1996). Misdiagnosis of symptomatic cerebral aneurysm. Prevalence and correlation with outcome at four institutions. *Stroke*.

[36] Liberman A. L., Gialdini G., Bakradze E., Chatterjee A., Kamel H., Merkler A. E. (2018). Misdiagnosis of cerebral vein thrombosis in the emergency department. *Stroke*.

[37] Hussain F., Cooper A., Carson-Stevens A. (2019). Diagnostic error in the emergency department: learning from national patient safety incident report analysis. *BMC Emergency Medicine*.

